# Association of the endothelial protein C receptor (*PROCR*) rs867186-G allele with protection from severe malaria

**DOI:** 10.1186/1475-2875-13-105

**Published:** 2014-03-17

**Authors:** Izumi Naka, Jintana Patarapotikul, Hathairad Hananantachai, Hiroo Imai, Jun Ohashi

**Affiliations:** 1 Faculty of Medicine, University of Tsukuba, Tsukuba, Ibaraki, Japan; 2 Faculty of Tropical Medicine, Mahidol University, Bangkok, Thailand; 3 Molecular Biology Section, Department of Cellular and Molecular Biology, Primate Research Institute, Kyoto University, Inuyama, Aichi, Japan

## Abstract

**Background:**

Cytoadhesion of *Plasmodium falciparum*-infected erythrocytes to endothelial cells in microvessels is a remarkable characteristic of severe malaria. The endothelial protein C receptor (EPCR), encoded by the endothelial protein C receptor gene (*PROCR*), has recently been identified as an endothelial receptor for specific *P. falciparum* erythrocyte membrane protein 1 (PfEMP1) subtypes containing domain cassettes (DCs) 8 and 13. The *PROCR* rs867186-G allele (serine-to-glycine substitution at position 219 of EPCR; 219Gly) has been shown to be associated with higher levels of plasma soluble EPCR (sEPCR). In this study, the association of *PROCR* rs867186 with severe malaria is examined in Thai population.

**Methods:**

A total of 707 Thai patients with *P. falciparum* malaria (341 with severe malaria and 336 with mild malaria) were genotyped for rs867186. To assess the association of *PROCR* rs867186 with severe malaria, three models (dominant, recessive and allelic) were evaluated. The rates of non-synonymous and synonymous substitutions were estimated for the coding sequence of the *PROCR* gene.

**Results:**

The rs867186-GG genotype was significantly associated with protection from severe malaria (*P*-value = 0.026; odds ratio = 0.33; 95% confidence interval = 0.12–0.90). Evolutionary analysis provided no evidence of strong positive selection acting on the *PROCR* gene.

**Conclusion:**

The rs867186-GG genotype showed significant association with protection from severe malaria. The present results suggest that PfEMP1–EPCR interaction, which can mediate cytoadhesion and/or reduce cytoprotective and anti-inflammatory effects, is crucial to the pathogenesis of severe malaria.

## Background

The cytoadhesion of *Plasmodium falciparum*-infected erythrocytes to endothelial cells in microvessels has been considered to be involved in the pathogenesis of severe malaria [1-3]. Several *P. falciparum* proteins (ligands) and human endothelial proteins (receptors) associated with the cytoadhesion have been identified to date [4-7]. The main parasite ligand expressed at the infected erythrocyte surface is a family of *P. falciparum* erythrocyte membrane protein 1 (PfEMP1) proteins. The PfEMP1 family is encoded by *var* genes, which are expressed in a mutually exclusive fashion [8]. The *var* gene family is classified into three main subgroups, A, B, and C. PfEMP1 variants encoded by group B and C *var* genes bind CD36, whereas non-CD36 binding variants are encoded by some group A var genes [9,10]. Recently, the PfEMP1 subtypes containing domain cassettes (DCs) 8 (group B/A hybrid) and 13 (group A) are shown to mediate adherence of *P. falciparum*-infected erythrocytes to brain endothelial cells [11,12]. A DC8-expressing clonal parasite line can also bind to primary microvascular cells from heart, lung, and dermis [11]. In addition, high transcription levels of genes encoding DC8 and DC13 are reported to be significantly associated with severe childhood malaria [13]. These observations suggest that DC8 and DC13 PfEMP1 variants play a key role in cytoadhesion of *P. falciparum*-infected erythrocytes to various endothelial cells. More recently, it has been demonstrated that the endothelial protein C receptor (EPCR) acts as an endothelial receptor for DC8 and DC13 PfEMP1 variants [7]. Of interest, binding to human brain microvascular endothelial cells via EPCR is significantly higher in parasite isolates from patients with severe malaria than in those from children with uncomplicated and mild malaria [7].

EPCR, a 46-kDa type 1 transmembrane glycoprotein, homologous to major histocompatibility complex class I/CD1 family proteins, is encoded by the endothelial protein C receptor gene (*PROCR*) (OMIM 600646) and is expressed on endothelial cells [14,15]. Endothelial cell-bound EPCR is a receptor for protein C and facilitates the activation of protein C. EPCR also exists as a soluble form of EPCR (sEPCR), resulting from metalloprotease-mediated EPCR membrane shedding [16], in human plasma. In contrast to endothelial cell-bound EPCR, sEPCR inhibits the activation of protein C by competing for protein C with endothelial cell-bound EPCR. Interestingly, variations in plasma sEPCR levels are mostly determined by the genotype of rs867186 (Ser219Gly), a non-synonymous single nucleotide polymorphism (SNP) of the *PROCR* gene [17-24]. The rs867186-G allele is strongly associated with increased sEPCR levels. The rs867186-G allele encodes glycine instead of serine at codon 219, which is located in the transmembrane region of EPCR. Therefore, rs867186 may also introduce conformational changes in endothelial cell-bound EPCR.

These observations raise the question of whether the *PROCR* rs867186 allele affects the pathogenesis of malaria through changes in the ability of PfEMP1 proteins with DC8 or DC13 to bind EPCR on endothelial cells. In this study, the association of rs867186 with severe malaria was examined in Thai adult patients with malaria. The results indicate that the rs867186-GG genotype is significantly associated with protection from severe malaria.

## Methods

### Subjects

A total of 707 adult patients with *P. falciparum* malaria living in Suan Phung, Ratchaburi-Province, Northwest Thailand were investigated in this study. All the patients underwent treatment at the Hospital for Tropical Diseases, Faculty of Tropical Medicine, Mahidol University, Bangkok, Thailand. Malarial infection by *P. falciparum* was confirmed in all the patients by a positive blood smear for the asexual form of *P. falciparum*. Clinical manifestations of malaria were classified according to the definitions and associated criteria of the World Health Organization. Severe malaria was defined as the presence of one of the following signs: high parasitaemia (>100,000 parasites/ml), hypoglycaemia (glucose level <22 nmol/l), severe anaemia (haematocrit <20% or haemoglobin level <7 g/dl), increased serum creatinine (>3 mg/dl), and unrousable coma caused by malaria infection. Cerebral malaria was defined as the presence of unrousable coma regardless of the other signs. Mild malaria was defined as having fever without other causes of infection and the absence of signs indicating severe malaria. According to the definition mentioned above, 707 adult malaria patients were classified into 341 patients with severe malaria (108 patients with cerebral malaria and 233 with non-cerebral severe malaria) and 366 with mild malaria. All the individuals were ≥13 years of age, and the mean age of patients with severe and mild malaria was 25.4 and 25.8 years, respectively. This study was approved by the institutional review board of the Faculty of Tropical Medicine, Mahidol University, and the Research Ethics Committee of the Graduate School of Comprehensive Human Sciences, University of Tsukuba, Tsukuba, Ibaraki, Japan. Written informed consent was obtained from all the patients. Three western chimpanzees (*Pan troglodytes verus*) were also investigated.

### DNA extraction

Genomic DNA was extracted from the peripheral blood leukocytes of patients with malaria and from peripheral blood samples of western chimpanzees using the QIAamp^®^ DNA Blood Kit (Qiagen GmbH, Hilden, Germany) and the DNeasy^®^ Blood & Tissue Kit (Qiagen GmbH), respectively.

### Sequencing analysis

Variations in the coding region of the *PROCR* gene were screened in seven patients with malaria (four with mild and three with severe malaria) and three western chimpanzees by direct sequencing. The seven patients with malaria were randomly selected from all the subjects studied. Primer sequences designed to cover the entire coding region of the *PROCR* gene (NM_006404.3) are presented in Table 1. Polymerase chain reaction (PCR) conditions are available upon request. PCR amplification was performed using the GeneAmp^®^ PCR System 9700 (Applied Biosystems, Foster City, CA, USA) and the FastStart Taq DNA Polymerase Kit (Roche Molecular Biochemicals, Mannheim, Germany). PCR products were sequenced using an ABI Prism^®^ 3100 Genetic Analyzer (Applied Biosystems).

**Table 1:** Primers used in this study

**Product**	**Primer name**	**Primer sequence**
PCR 1	PCR 1 F	GAGAAGGGAAAAGGCAGGTC
	PCR 1 R	TGCCTGCCCTGTAGAGAGAT
PCR 2	PCR 2 F	CCTCGAGGTAGGGGGTTATT
	PCR 2 R	CACCCAGCAATCTTCAAAGG
PCR 3	PCR 3 F	TCATGTTCTTTTCCCCTTGG
	PCR 3 R	CCATCCATTTGTCTGGAACC
PCR 4-1	PCR 4–1 F	CACACGCAGCTTCAGTCAGT
	PCR 4–1 R	TCCCATCCCAAGTCTGACAC
PCR 4-2	PCR 4–2 F	TGGCCCATCCTCCAAAGACAG
	PCR 4–2 R	CCAGAAATTTTGCAAAGTGGA

### Genotyping

The *PROCR* rs867186 SNP was genotyped using the TaqMan^®^ SNP genotyping assay.

### Statistical analysis

The Fisher’s exact test based on a 2 × 2 table was used to assess the association of rs867186 with susceptibility to severe malaria. In the association test, three models (dominant, recessive and allelic) with regard to rs867186-G were examined. The significance level of this study was set to be 0.05 in two-sided test. The individual genotypes of SNPs in HapMap-CHB (Han Chinese in Beijing) and HapMap-JPT (Japanese in Tokyo) populations were retrieved from the HapMap database [25,26] and 1,000 genome project database [27], and then pair-wise linkage disequilibrium (LD) parameters, *r*^2^, between *PROCR* SNPs were estimated by using Haploview software [28]. The PolyPhen-2 (Polymorphism Phenotyping v2) software [29] was used to predict possible impact of the serine-to-glycine substitution at codon 219 (i e, rs867186-A to -G) on the structure and function of EPCR. The difference in transcription factor DNA-binding specificity between two alleles at each SNP was evaluated using the RegSNP [30], a web tool for predicting the effect of SNP on transcription factor-DNA binding. The number of nucleotide substitutions per site between two individual sequences was estimated for the *PROCR* coding region, based on the two-parameter method of Kimura [31]. Based on the estimated number of substitutions, a phylogenetic tree was constructed by using the neighbour-joining method [32] as implemented in MEGA 5.0 [33]. Tajima’s relative rate test [34,35] was performed using MEGA 5.0 software [33]. The *PROCR* mRNA sequences of rhesus macaque (GenBank Accession No. XM_001100647) was retrieved from the GenBank database, and used as an outgroup sequence. For *PROCR* coding sequences of human and chimpanzee, the number of non-synonymous substitutions per non-synonymous site (*d*_N_) and the number of synonymous substitutions per synonymous site (*d*_S_) were estimated using the Nei–Gojobori method with the Jukes–Cantor model [36]. The variance of (*d*_N_ - *d*_S_) was estimated using the bootstrap resampling method (1,000 resamplings), and *P*-value based on Z-test statistics were calculated using MEGA 5.0 software [33].

## Results

### Screening for variation

**Figure 1:**
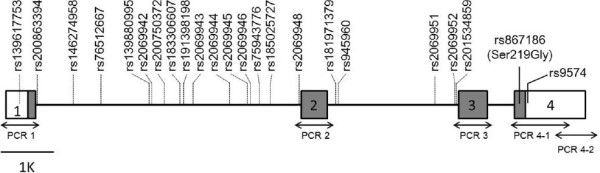
***PROCR *single nucleotide polymorphism detected in this study.** A total of 23 *PROCR* SNPs were polymorphic in a sample set of 97 CHB subjects and 89 JPT subjects in the 1000 Genomes Project database. Two SNPs (rs867186 [Ser219Gly] and rs9574) indicated by solid lines were detected in a variation screening for seven malaria subjects. The PCR fragments investigated in this study are shown below the structure of the *PROCR* gene (NM_006404.3). The open and shaded boxes indicate UTR and coding regions, respectively. The numbers in the boxes indicate the exon number of the *PROCR* gene.

Screening for variation in the entire coding region of *PROCR* detected one non-synonymous SNP (rs867186 [Ser219Gly]) in exon 4 and one SNP (rs9574) in 3′UTR in seven Thai patients with malaria, which suggests that there was no common non-synonymous SNP other than rs867186 in the Thai population. To confirm this, polymorphisms of the *PROCR* gene in Asian populations were searched using the 1000 Genomes Project database [27]. In addition to rs867186 and rs9574, 21 *PROCR* SNPs were polymorphic in a sample set of 97 CHB subjects and 89 JPT subjects: one SNP in 5′UTR and 20 SNPs in introns (Figure 1). Of 23 SNPs detected in the 1000 Genomes Project database, 10 SNPs (rs867186, rs9574, and eight intronic SNPs) had a minor allele frequency of >0.05. Each of these eight intronic SNPs was tagged by either rs867186 or rs9574 with an *r*^2^ threshold of 0.8. These results suggest that the *PROCR* gene has only one common non-synonymous SNP, rs867186, and exhibits limited haplotype diversity in Asian populations. Thus, in this present study, rs867186, which has been shown to have functional significance [17-24], was investigated.

### Association test

The genotype frequencies of rs867186 in patients with malaria are shown in Table 2. A comparison of the nucleotide sequence of human *PROCR* with that of the western chimpanzee indicated that rs867186-G (219Gly) is a derived allele (a point mutation that occurred in the human lineage). Therefore, the direction of association was determined by rs867186-G. Table 3 shows the results from association tests based on three models (dominant, recessive and allelic). A recessive model (rs867186-GG *versus* rs867186-AG and rs867186-AA) showed a statistically significant difference in genotype frequency between patients with severe and mild malaria (*P*-value = 0.026; odds ratio [OR] = 0.33; 95% confidence interval [CI] = 0.12–0.90). Thus, rs867186-GG was associated with protection from severe malaria.

**Table 2:** Genotype frequencies of rs867186 in Thai malaria patients

**Genotype**	**Severe malaria**	**Mild malaria**
	**n (frequency)**	**n (frequency)**
GG	5 (0.015)	16 (0.044)
GA	94 (0.276)	88 (0.240)
AA	242 (0.710)	262 (0.716)

**Table 3:** Association of rs867186 with severe malaria

**Model**	***P*-value**	**OR**	**95% CI**
Dominant (GG + GA vs AA)	0.87	1.03	0.74-1.43
Recessive (GG vs GA + AA)	0.026	0.33	0.12-0.90
Allele (G vs A)	0.56	0.92	0.69-1.22

The *P*-value was calculated by Fisher’s exact test.

### *In silico* analysis of rs867186

**Figure 2:**
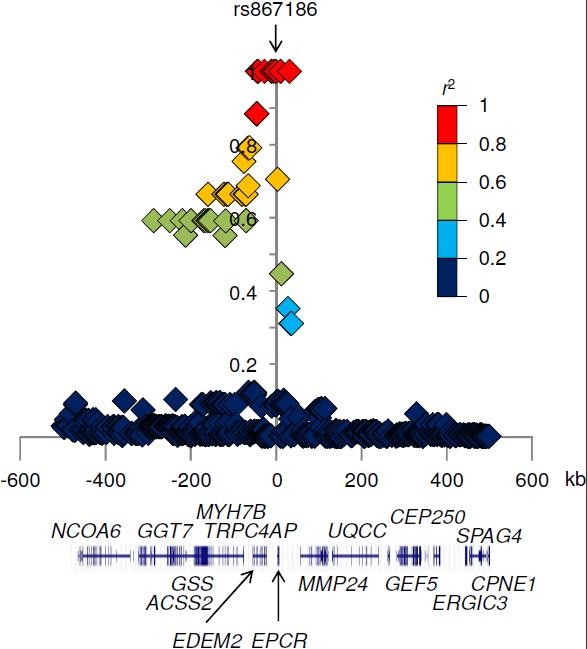
**Plot of linkage disequilibrium between rs867186 and nearby single nucleotide polymorphism.** LD coefficient *r*^2^ between rs867186 and each of 553 SNPs with minor allele frequency of more than 0.03 in the HapMap-CHB and -JPT populations were plotted. Each plot is coloured based on the value of *r*^2^.

A serine-to-glycine substitution at codon 219 of EPCR (i e, from rs867186-A to rs867186-G) was predicted to be “benign”, with a score of 0.201, by PolyPhen-2 software. Although rs867186-G was statistically associated with protection from severe malaria, rs867186 may not be an SNP primarily associated with severe malaria. In other words, the significant association of rs867186 with severe malaria may come from LD between rs867186 and a causative SNP. Analysis of LD detected ten SNPs in strong LD (*r*^2^ ≥ 0.8) with rs867186 in Asian populations (HapMap-CHB + JPT; Figure 2). Of these, six SNPs, rs6120843, rs1033797, rs1033799, rs11908683, rs2295888, and rs11907010, are located in the genomic region of the endoplasmic reticulum (ER) degradation enhancer, mannosidase alpha-like 2 gene (*EDEM2*; Figure 2). EDEM2 is involved in the ER-associated degradation of glycoproteins [37]. Because EDEM2 does not appear to be involved in malaria pathogenesis, the six *EDEM2* SNPs would not be primarily associated with severe malaria. The other four SNPs were located closer to the *PROCR* gene. Two of these, rs8119351 and rs2069940, are located in the upstream region of *PROCR*. However, no difference in transcription factor–DNA-binding specificity between two alleles at each of these SNPs was predicted by RegSNP [30]. The remaining two SNPs, rs11167260 and rs17092456, are located more than 10 kb downstream of the coding region of *PROCR*; thus, these SNPs are unlikely to affect its transcription.

### Evolutionary analysis

A phylogenetic tree was constructed on the basis of the estimated number of nucleotide substitutions in the coding region of *PROCR* (Additional file 1). A comparison of human, chimpanzee and macaque sequences suggested that a synonymous substitution had been fixed in the human lineage after the divergence between humans and chimpanzees, whereas four substitutions (three non-synonymous and one synonymous) had been fixed in the western chimpanzee lineage (Additional file 2). To examine whether the substitution rate is different between human and chimpanzee, Tajima’s relative rate test was performed. The result revealed no significant difference in the nucleotide substitution rate between humans and chimpanzees.

The observation that EPCR acts as a receptor for DC8 and DC13 PfEMP1 [7] raises the question of whether the human *PROCR* gene has been subjected to positive selection. An excess in the non-synonymous substitution rate relative to the synonymous rate when compared to neutral expectations is regarded as the signature of positive selection. To assess the possibility of natural selection acting on *PROCR* after the divergence between humans and chimpanzees, the number of non-synonymous substitutions per non-synonymous site and the number of synonymous substitutions per synonymous site were calculated for the human and chimpanzee coding sequences of *PROCR*. The rate of non-synonymous substitution (*d*_N_ = 0.006 for 219Ser and *d*_N_ = 0.008 for 219Gly) did not significantly differ from that of synonymous substitution (*d*_S_ = 0.011), suggesting that *PROCR* has not been under strong positive selection. It is noted here that the values of *d*_N_ were calculated for human *PROCR* bearing 219Ser and 219Gly, separately.

## Discussion

The *PROCR* rs867186 was significantly associated with severe malaria in Thai adult patients. There are two possible explanations for the association of rs867186-GG with protection from severe malaria. The serine-to-glycine substitution at codon 219 in the transmembrane of EPCR may introduce a conformational change in the molecule, which prevents DC8 and DC13 PfEMP1 from binding EPCR. The rs867186-G is associated with higher levels of sEPCR [17-24], and the addition of recombinant sEPCR inhibits the binding between DC8-expressing parasites and human brain microvascular endothelial cells [7]; therefore, the other possible explanation is that higher levels of sEPCR in malaria patients with the rs867186-GG genotype cause greater inhibition of the binding of DC8 and DC13 PfEMP1 to EPCR on endothelial cells through preferential binding of DC8 and DC13 PfEMP1 to sEPCR compared with EPCR. In this case, malaria patients with the rs867186-GG genotype may efficiently block the cytoadhesion of *P. falciparum*-infected erythrocytes to the endothelial cells of microvessels. In addition, if DC8 and DC13 PfEMP1 variants are more likely to bind sEPCR than protein C, more protein C may be allowed to bind EPCR on endothelial cells in malaria patients with the rs867186-GG genotype than in those with the other genotypes. Binding of protein C to EPCR leads to the activation of protein C. Activated protein C exerts cytoprotective and anti-inflammatory effects, which may contribute to protection against severe malaria [7], including cerebral malaria [38].

EPCR binding is significantly higher in parasite isolates from African children with severe malaria than in those from children with uncomplicated and mild malaria [7], suggesting that binding of PfEMP1 to EPCR may be a common risk factor of severe malaria regardless of its form. Cerebral malaria, a form of severe malaria that involves encephalopathy, is considered to be caused by excessive adherence of *P. falciparum*-infected erythrocytes to the microvasculature of the brain. In this study, among 341 severe malaria patients, 108 patients had suffered from cerebral malaria. When patients with severe malaria were divided into two groups: 108 patients with cerebral malaria and 233 patients with non-cerebral severe malaria, rs867186-GG was found to be significantly associated with protection from non-cerebral severe malaria (*P*-value = 0.014; OR = 0.19; 95% CI = 0.04–0.83), but not with cerebral malaria (*P*-value = 0.59; OR = 0.63; 95% CI = 0.18–2.19) (Additional file 3). The lack of association with cerebral malaria may have resulted from the small sample size (i.e., 108 cerebral malaria patients), since the direction of association observed for cerebral malaria was same as non-cerebral severe malaria (i.e., ORs for cerebral malaria and non-cerebral severe malaria were smaller than 1). In addition, misclassification of cerebral malaria (i.e., some cerebral malaria patients may have had coma without cerebral sequestration) may have reduced the statistical power of the present study. Considering the accumulated evidence that the DC8 and DC13 PfEMP1 variants mediate adherence of *P. falciparum*-infected erythrocytes to brain endothelial cells [11,12], the possibility of rs867186-GG being associated with protection from cerebral malaria should not be excluded. Studies with a larger number of cerebral malaria patients will be needed to examine the possible association of rs867186 with cerebral malaria.

If the binding of DC8 and DC13 PfEMP1 to EPCR on brain endothelial cells plays a crucial role in cerebral malaria, lower expression of EPCR is expected to decrease the risk for cerebral malaria. However, a recent study has shown that endothelial EPCR expression is significantly decreased in cerebral blood vessels from Malawian children with cerebral malaria characterized by sequestration of *P. falciparum*-infected erythrocytes than in controls characterized by absence of *P. falciparum*-infected erythrocyte sequestration [39]. To further understand the role of EPCR in the pathogenesis of cerebral malaria, levels of endothelial expression of cell-bound and soluble forms of EPCR are required to be compared between cerebral and mild malaria patients, after adjustment for rs867186 genotype and expression levels of DC8 and DC13 PfEMP1 variants.

No evidence of positive selection was found in the evolutionary analysis based on the comparison of *d*_N_ with *d*_S_. There are a few possible explanations: the first is that the statistical power was low. Only five substitutions had been fixed either in the human lineage or in the chimpanzee lineage after the divergence between them. The second is that the number of target sites (codons) of positive selection may be small. If most sites are under purifying selection, it would not be possible to detect the significant difference between *d*_N_ with *d*_S_ in the entire coding region. The third is that PfEMP1 subtypes containing DC8 and DC13 may have appeared recently in *P. falciparum* and advantageous mutations that help to escape from the binding of DC8 and DC13 PfEMP1 to EPCR have not occurred yet in human *PROCR*.

## Competing interests

The authors declare that they have no competing interests.

## Authors’ contributions

IN and JO performed statistical analyses and wrote the manuscript. IN performed PCR direct sequencing and genotyping. IN, JP and HH extracted DNA from human blood samples. HI extracted DNA from western chimpanzee blood samples. IN, JP, HI, and JO participated in the design and coordination of the study. All the authors read and approved the final manuscript.

## Supplementary Material

Additional file 1 **A phylogenetic tree of *PROCR*.** Based on the estimated number of nucleotide substitutions, a phylogenetic tree of 14 human sequences, six western chimpanzee sequences, and a macaque sequence of the coding region of *PROCR* was constructed using the neighbour-joining method. The scale bar indicates nucleotide substitutions per site. Click here for file

Additional file 2 **Multiple sequence alignment of the coding region of *PROCR*.** Click here for file

Additional file 3 Genotype frequencies of rs867186 in cerebral, non-cerebral severe, and mild malaria patients. Click here for file
