# Supplementary material for: Association of the endothelial protein C receptor (PROCR) rs867186-G allele with protection from severe malaria
Source: Malar J. 2014 Mar 17;13:105. doi: 10.1186/1475-2875-13-105 (PMC4004250; doi:10.1186/1475-2875-13-105)
Supplement: Additional file 1 — A phylogenetic tree of PROCR. Based on the estimated number of nucleotide substitutions, a phylogenetic tree of 14 human sequences, six western chimpanzee sequences, and a macaque sequence of the coding region of PROCR was constructed using the neighbour-joining method. The scale bar indicates nucleotide substitutions per site. [file 1475-2875-13-105-S1.pptx]

## Slide 1
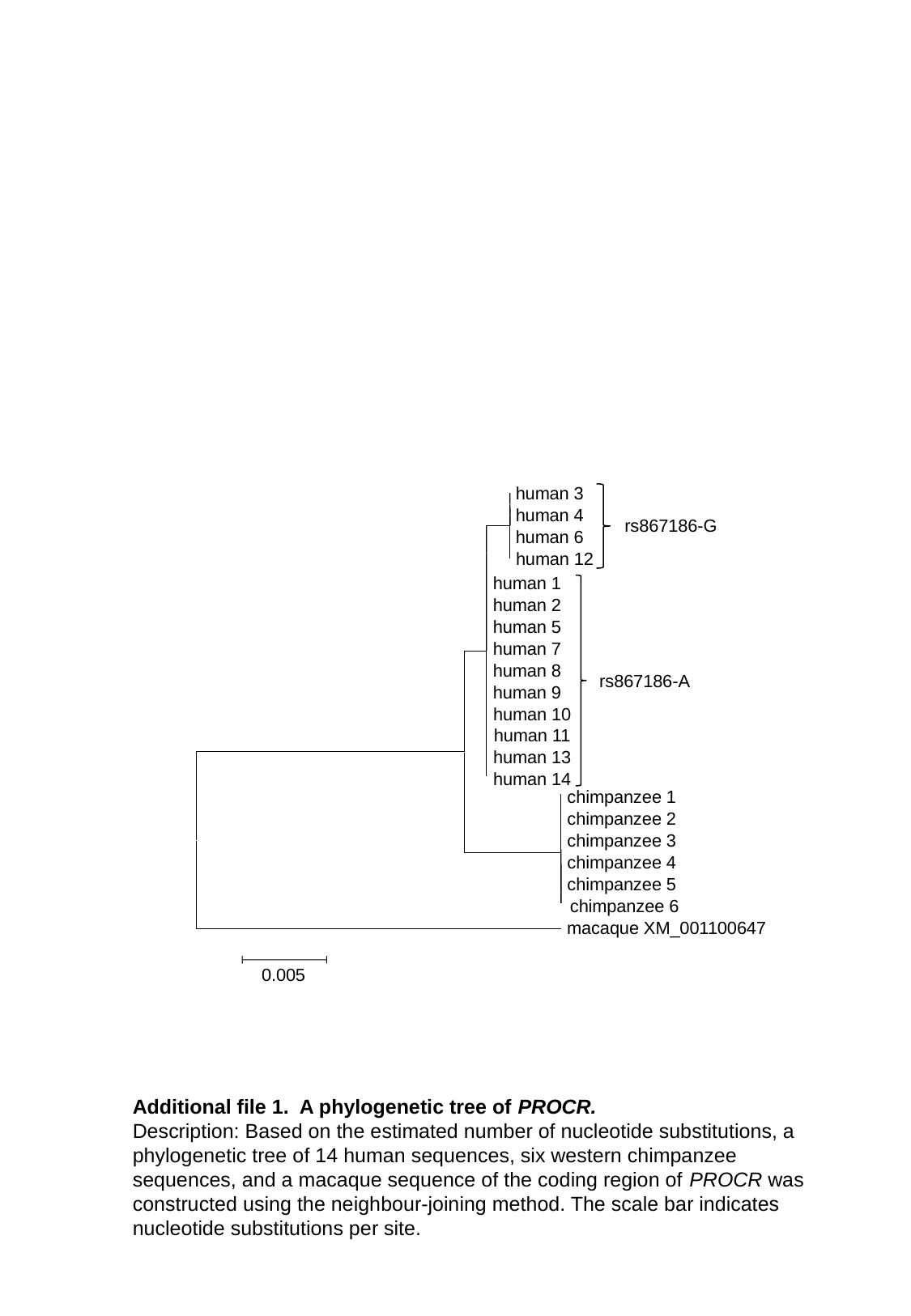

human 3
 human 4
rs867186-G
 human 6
 human 12
 human 1
 human 2
 human 5
 human 7
 human 8
rs867186-A
human 9
 human 10
 human 11
 human 13
 human 14
 chimpanzee 1
 chimpanzee 2
 chimpanzee 3
 chimpanzee 4
 chimpanzee 5
 chimpanzee 6
macaque XM_001100647
0.005
Additional file 1. A phylogenetic tree of PROCR.
Description: Based on the estimated number of nucleotide substitutions, a phylogenetic tree of 14 human sequences, six western chimpanzee sequences, and a macaque sequence of the coding region of PROCR was constructed using the neighbour-joining method. The scale bar indicates nucleotide substitutions per site.
