# Supplementary figures and images for: Association of the endothelial protein C receptor (PROCR) rs867186-G allele with protection from severe malaria
Source: Malar J. 2014 Mar 17;13:105. doi: 10.1186/1475-2875-13-105 (PMC4004250; doi:10.1186/1475-2875-13-105)

## Slide 1
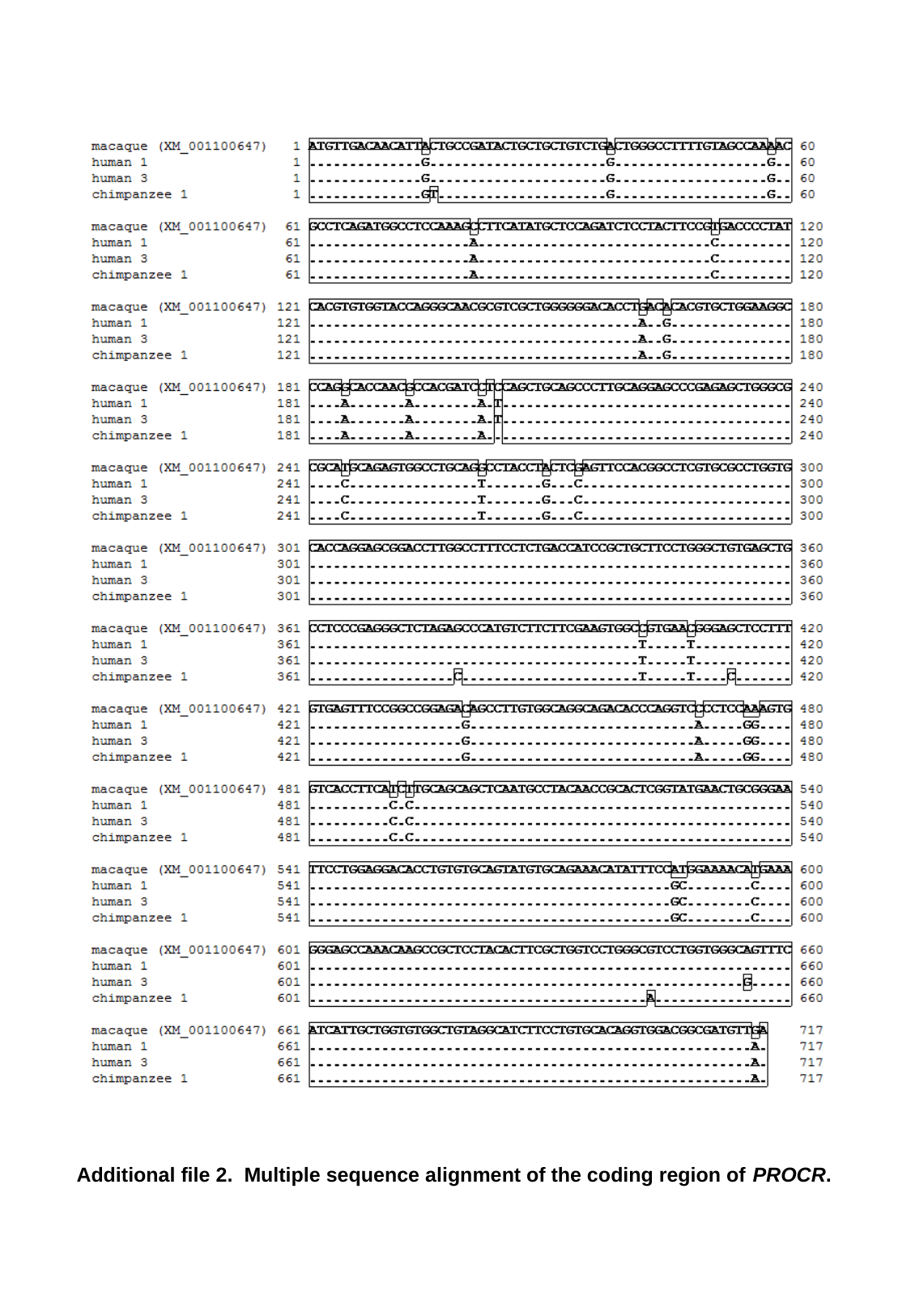

Additional file 2. Multiple sequence alignment of the coding region of PROCR.

Supplement: Additional file 2 — Multiple sequence alignment of the coding region of PROCR . [file 1475-2875-13-105-S2.pptx]
